# *Evolvulus alsinoides *methanolic extract triggers apoptosis in HepG2 cells

**Published:** 2018

**Authors:** Murugan Ponselvi Induja, Devaraj Ezhilarasan, Nandigam Ashok Vardhan

**Affiliations:** 1 *Department of Pharmacology, Biomedical Research Unit and Laboratory Animal Centre (BRULAC), Saveetha Dental College and Hospitals, Saveetha Institute of Medical and Technical Sciences, Saveetha University, Chennai, Tamil Nadu, India*; 2 *Department of Biochemistry, Biomedical Research Unit and Laboratory Animal Centre (BRULAC), Saveetha Dental College and Hospitals, Saveetha Institute of Medical and Technical Sciences, Saveetha University, Chennai, Tamil Nadu, India *

**Keywords:** Apoptosis, Dual staining, Cytotoxicity, Proliferation, Catenin – β 1

## Abstract

**Objective:**

The objective of the present study was to evaluate the cytotoxic potentials of *Evolvulus alsinoides *in human hepatoma HepG2 cells.

**Materials and Methods:**

HepG2 cells were treated with methanolic extract of *E. alsinoides* at 20, 40 and 80 µg/ml for 24 hr and cytotoxic effect was analyzed by MTT assay. The apoptosis rate was investigated by Hoechst 33342 and annexin V/propidium iodide staining. Mitochondrial membrane potential was evaluated by rhodamine staining. Also, the expression of catenin – β 1 protein was analyzed by western blotting.

**Results:**

*E. alsinoides *methanolic extract treatment caused significant cytotoxicity in HepG2 cells in a concentration-dependent manner. Dual staining assay confirmed the presence of early and late apoptotic cells only in extract-treated groups. Plant extract treatment also caused nuclear fragmentation and chromatin condensation in HepG2 cells. Mitochondrial membrane potential also reduced upon *E.* *alsinoides *treatments. This treatment also modulated the catenin – β 1 protein expression.

**Conclusion:**

In this study, we demonstrated the proapoptotic potential *E.* *alsinoides *in HepG2 cells; thus, this plant may be beneficial in the treatment of liver cancer.

## Introduction

Hepatocellular carcinoma (HCC) is the most frequent primary liver malignancy and is a leading cause of cancer-related death, worldwide (Hartke et al., 2017 ▶). Chronic hepatitis B and C virus infections, alcohol drinking, tobacco smoking, obesity, and diabetes are the most common risk factors predisposing to HCC development (Baecker et al., 2018 ▶). The current treatment modalities for HCC include surgical ablation and liver transplantation (Thandassery et al., 2014 ▶). Multiple treatment regimens using chemotherapeutic drugs exist with differing advantages and disadvantages. For instance, sorafenib, a mitogen-activated protein (MAP) kinase pathway inhibitor, is currently used to improve the survival of HCC patients; however, this medication has several adverse effects such as hyperbilirubinemia, hand and foot skin reactions and fatigue (Agrawal et al., 2016 ▶; Salhab and Canelo, 2011 ▶). Despite significant advancement in HCC management, its incidence continues to rise (Arora and Kumar, 2014 ▶). Therefore, development of therapeutic modalities for the management of HCC with minimal or no side effects and is need of the hour. Medicinal plants and derived phytoconstituents play an imperative role in drug development and are being increasingly documented as useful components of complementary alternative medicine for cancer therapy (Ezhilarasan, 2018 ▶). Accumulating evidence concretely indicates the beneficial effect of herbal medicines on the improvement of survival rate and quality of life, along with immunomodulatory effects in cancer patients (Yin et al., 2013 ▶). Additionally, it must be noted that herbal medicines are being used in combination with conventional anticancer drugs as adjuvants (Yue et al., 2017 ▶). 

*Evolvulus alsinoides* L. is a medicinal plant used in Indian traditional system of medicine for preparation of 'Shankhpushpi', an important and popular ayurvedic drug that considerably contributes to the improvement of memory (Sharma et al., 2016 ▶). *E. alsinoides* is reported to have myriad beneficial effects such as neuroprotective (Naikawadi et al., 2016 ▶), antioxidative (Gomathi et al., 2014 ▶; Kumar et al., 2010 ▶), anti-diabetic (Gomathi et al., 2013 ▶) immunomodulatory (Ganju et al., 2003 ▶) properties. In previous studies, GC-MS analysis revealed the presence of several promising active phytocompounds like piperine, octadecanoic acids, hexadecanoic acid and squalene in the ethanolic extract of *E. alsinoides* (Gomathi et al., 2015 ▶). Therefore, it is reasonable to assume that *E. alsinoides* may have anticancer activities due to the presence of secondary metabolites. Though *E. alsinoides *has beneficial effects against several diseases, till date, its cytotoxic potential has not been reported against any cancer cell line. Hence, in this study, we evaluated the cytotoxic potential of *E. alsinoides *in human hepatoma HepG2 cell line.

## Materials and Methods

**Reagents**

Dulbecco’s minimum essential low glucose medium (DMEM), penicillin, streptomycin, tryspin-EDTA, 3-(4, 5-dimethylthiazol-2-yl)-2, 5-diphenyltetrazolium bromide (MTT), and fetal bovine serum (FBS) were obtained from GIBCO BRL (Gaithersburg, MD). All other chemicals used in this study were of analytical grade.

**Collection of plant material and extraction**

*E. alsinoides *whole plants were collected in June to September in 2017 from Hyderabad, Telangana, India and authenticated by the Department of Plant Genetics, Osmania University, Hyderabad, Telangana, India. The whole plants were shade-dried and powdered; then, 10 g of the powder was extracted by methanol using Soxhlet apparatus. The crude extracts were concentrated under reduced pressure and the methanolic extract powder of *E. alsinoides* was used in the present study. 

**Cell cultures and treatment**

The HepG2 human hepatocellular carcinoma cell line was procured from NCCS with the passage number 17. Cells were maintained in DMEM with low glucose supplemented with 10% FBS, 100 units/mL penicillin and 100 μg/mL streptomycin. Cells were cultured in a humidified atmosphere with 5% CO_2_ at 37°C. Cells were grown in 25-cm^2^ culture flasks and after a few passages, they were seeded for experiments. The experiments were done at 70 to 80% confluence. Upon reaching confluence, cells were detached using a 0.25% trypsin-EDTA solution.

**Figure:**
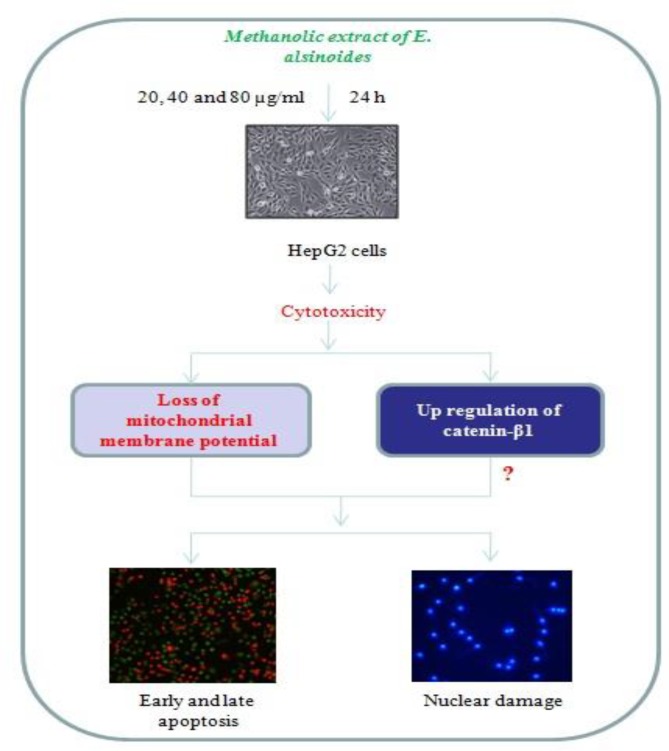


HepG2 cells were plated at 1×10^4^ cells/cm^2^. After 24 hr, cells were fed with fresh expansion culture medium supplemented with different final concentrations of *E. alsinoides* (20, 40 and 80 µg/ml) or the corresponding volumes of vehicle (0.1% dimethyl sulfoxide (DMSO)). After 24 hr of treatment, cells were collected by trypsin application. Total cell number was determined by counting each sample in triplicate under an inverted microscope. 

**MTT assay**

The cytotoxic property of E. alsinoides was evaluated using MTT assay (Safadi et al., 2003). Cells were plated in a 96-well plate at a concentration of 1×10^4^ cells/well. After 24 hr, the medium was replaced with 100 µl of medium containing *E. alsinoides* extract at different concentrations and incubated for 24 hr. At the end of the treatment period, media from control, and *E. alsinoides-*treated cells were discarded and 50 μl of MTT (5 mg/ml in phosphate buffered saline (PBS) solution was added to each well. Cells were then incubated for 4 hr at 37°C in CO_2_ incubator. MTT was then aspirated and the formazan crystals were dissolved in 150 μl of DMSO. The purple blue formazan formed was measured using an ELISA reader (BIORAD) at 570 nm. Optical density of each sample was compared with that of the control and graphs were plotted. 

**Detection of apoptosis via AnnexinV/propidium iodide (PI) staining**

Morphological changes related to apoptosis were analyzed through annexin V and PI staining (Baskić et al., 2006 ▶). HepG2 cells were plated at a density of 1×10^4^ in 48-well plates. They were allowed to grow at 37°C in a humidified CO_2_ incubator until they reached 70-80% confluence. Then, cells were treated with *E. alsinoides *for 24 hr. afterwards, the culture medium was aspirated from each well and cells were gently rinsed twice with PBS at room temperature. The cells were collected from control and experimental groups then re-suspended in PBS and incubated with annexin V reagent in 4-(2-hydroxyethyl)-1-piperazineethanesulfonic acid (HEPES) buffer containing PI and viewed immediately under a Nikon inverted fluorescence microscope (Japan, Ti series). 

**Analysis of nuclear chromatin condensation by Hoechst staining**

HepG2 cells (2 × 10^4^ cells/well) were seeded into a 96-well and treated with different concentrations of *E. alsinoides* for 24 hr at 37˚C. The cells were washed three times with PBS and incubated with 50 µl Hoechst 33342 solution (100 µg/ml) at room temperature for 10 min. The cells were washed twice in PBS, and then cells morphology was observed immediately under a Nikon inverted fluorescence microscope (Japan, Ti series) (Crowley et al., 2016 ▶) 

**Analysis of mitochondrial membrane potential by rhodamine staining assay**

Mitochondria were stained with rhodamine (a red-fluorescent stain) which accumulates in mitochondria depending on the mitochondrial membrane potential. After 24 hr of treatment, cells were suspended in a 10 mM HEPES buffer (pH 7.4), containing 5% glucose, and rhodamine (10 μM stock solutions dissolved in DMSO) was added to a make final concentration of 100 nM. After 15 min of incubation, mitochondrial membrane potential was visualized by confocal microscopy at λex=555 nm and λem=579 nm (Yang et al., 1997 ▶). 

**Western blot analysis**

HepG2 cells were lysed in radioimmunoprecipitation assay buffer (20 mM tris-HCl, pH 7.6, 150 mM NaCl, 1% NP-40, 0.1% SDS, 1% deoxycholate sodium and 0.1% protease and phosphatase inhibitor cocktails. After extraction, protein concentration was estimated using bovine serum albumin (BSA) as the standard. Total protein extracts were subjected to sodium dodecyl sulfate-polyacrylamide gel electrophoresis and electroblotted onto nitrocellulose membranes. The membranes were blocked by 5% BSA, and incubated overnight at 4°C with catenin β-1 primary antibodies (monoclonal) and 2 hr at room temperature with corresponding secondary antibodies. Immunoreactive bands were detected by enhanced chemiluminescence with protein A-horseradish peroxidase and the SuperSignal chemiluminescent system and quantified as reported by Ezhilarasan et al. (2017) ▶.

**Statistical analysis**

Statistical analysis was performed using Graph Pad prism 5.0. Data were expressed as mean±SEM and analyzed by One way ANOVA followed by Dunnett’s test to compare differences among groups. A p<0.05 was considered to be significant.

## Results

***E. alsinoides*** **inhibited HepG2 cells proliferation**

**Figure 1:**
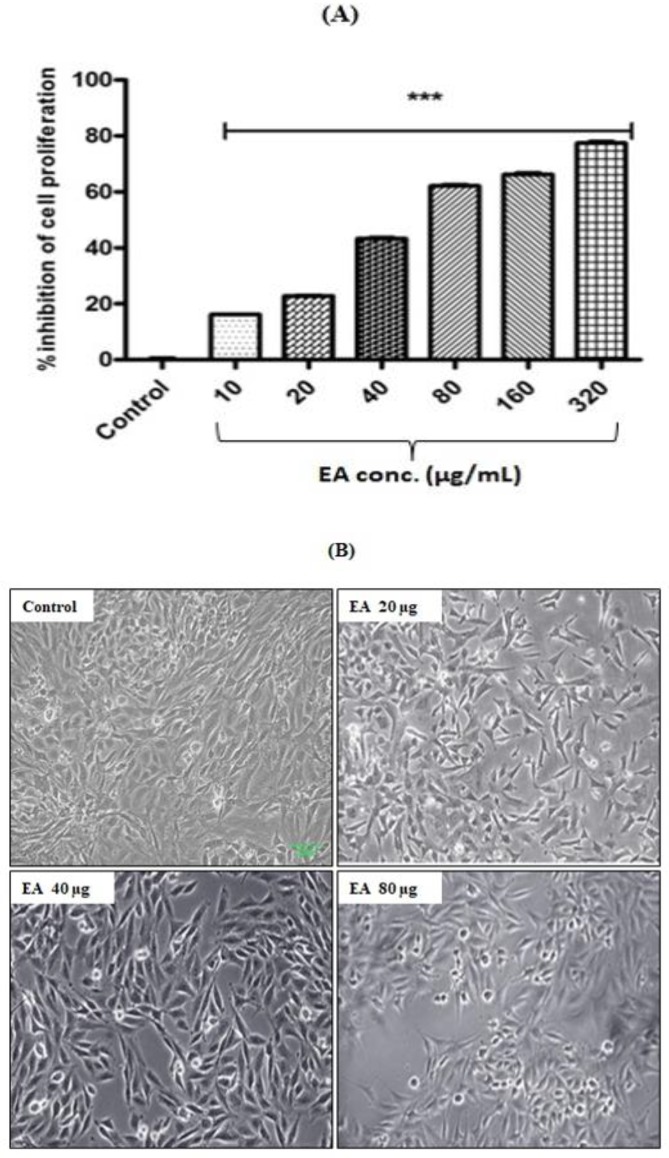
(A) Cytotoxic effect of *E. alsinoides* (EA) in HepG2 cells after 24 hr. Values are expressed as mean±SEM (n=3). ***p<0.001 shows significant differences as compared to control. (B) Morphology of HepG2 cells treated with EA methanolic extract

Initially, we attempted to find the effective concentration range of *E. alsinoides* that inhibits the proliferation of HepG2 cells. To find out the IC_50_ of *E. alsinoides*, HepG2 cells were treated with 10, 20, 40, 80, 160 and 320 µg/ml. *E. alsinoides* treatments for 24 hr caused a significant (p<0.001) and concentration-dependent decrease in the proliferation of HepG2 cells (Figure 1A) and the IC_50 _was 80 µg/ml. Hence, further studies were carried out by using doses below the IC_50_ (*i.e.,* 20, 40 and 80 µg/ml). The morphology of HepG2 cells treated with *E. alsinoides* is presented in Figure 1B. 

**Apoptosis analysis by annexin V and propidium iodide**

**Figure 2:**
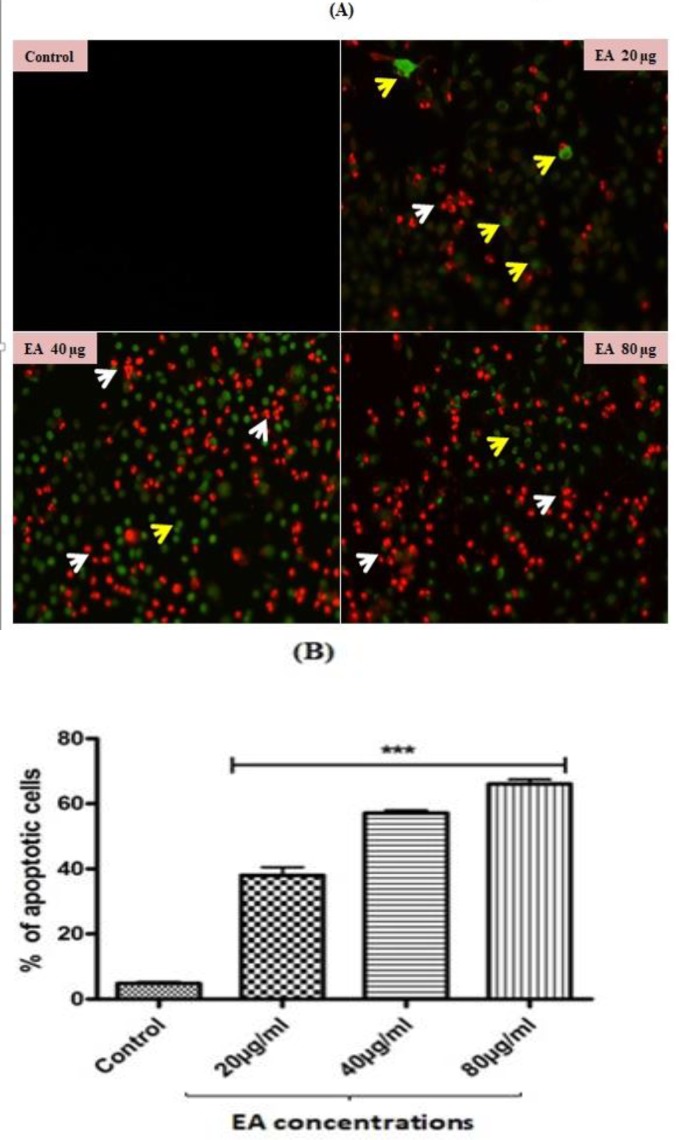
(A) Representative images of HepG2 cells (control and *E.alsinoides* (EA) treated cells) stained with annexin V and propidium iodide staining (20X). The yellow and white arrow heads shows early and late apoptotic HepG2 cells (B) Quantification of the percentage of apoptotic HepG2 cells. Values are expressed as mean±SEM (n=3). ***p<0.001 shows significant differences as compared to control

Annexin V and propidium iodide dual staining was used to morphologically distinguish early and late apoptotic cells. Presence of green color cells stained with annexin V conjugated with FITC confirmed the occurrence of early apoptosis in all experimental groups. Following treatment with *E. alsinoides*, HepG2 cell nuclei were stained with PI (as reflected in red) indicating late apoptosis (Figure 2A). The population of apoptotic HepG2 cells was significantly (p<0.001) increased upon *E. alsinoides* treatment (Figure. 2B).

**Chromatin condensation and nuclear fragmentation analysis by Hoechst Staining**

**Figure 3:**
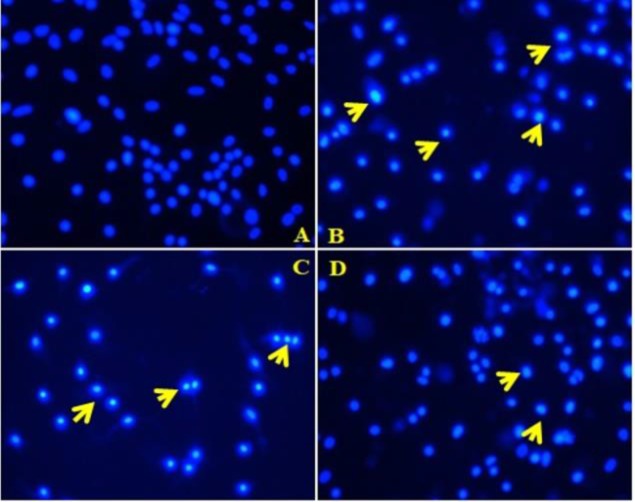
Nuclear morphology analysis by Hoechst staining (20X). A: control, and B, C and D: HepG2 cells treated with 20, 40 and 80 µg of *E. alsinoides*, respectively. Arrow heads indicate fragmented nuclei with condensed chromatin

Further, to gain more insight into the apoptosis-inducing potential of *E. alsinoides* in HepG2 cells, we analyzed morphological features of apoptotic nuclei. *E. alsinoides* treatments in HepG2 cells caused significant changes in the nucleus as evidenced by Hoechst staining. *E. alsinoides*-treated cells showed bright fluorescence which indicated the condensed chromatin and fragmented nuclei when compared to control cells (Figure 3). 

**EA decreases mitochondrial membrane potential**

**Figure 4:**
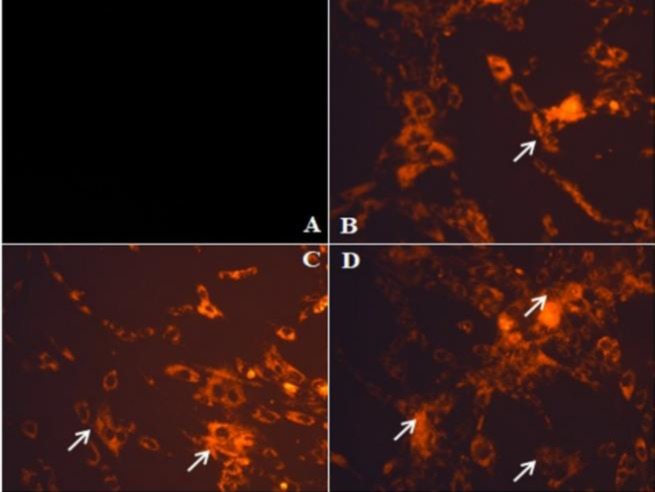
Analysis of mitochondrial membrane potential by rhodamine staining (40X). Arrows show loss of mitochondrial potential. A: control, and B, C and D: HepG2 cells treated with 20, 40 and 80 µg of *E. alsinoides*, respectively

To observe whether the release of cytochrome c from mitochondria is a consequence of depolarization, we treated HepG2 cells with different concentrations of *E. alsinoides* for 24 hr and the mitochondrial membrane potential (MMP) was evaluated by rhodamine 123 staining. The rhodamine 123 uptake was visualized by confocal microscopy. The HepG2 cells treated with *E. alsinoides* for 24 hr, showed significant loss of MMP. The most prominent effect was observed with the maximum concentration used in this study (Figure 4).

**Effect of** ***E. alsinoides*** **on catenin β-1 (CTNNB1) protein expression**

**Figure 5:**
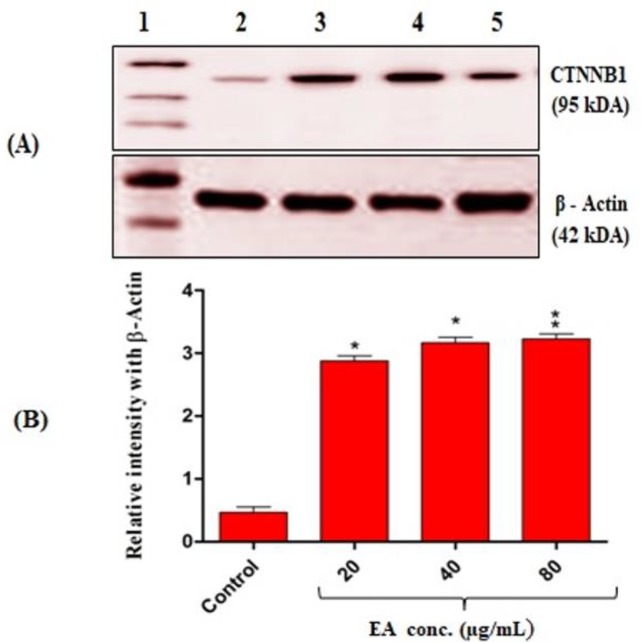
(A) Expression level of catenin-β1 protein as assessed by western blotting. 1: protein ladder, 2: control, 3, 4 and 5: HepG2 cells treated with 20, 40 and 80 µg of *E. alsinoides*, respectively. (B) Quantification of catenin-β1 protein expression. Values are expressed as mean±SEM (n=3). * p<0.05 and ** p<0.01 show significant differences as compared to control

Further, to analyze whether CTNNB1 is responsible for *E. alsinoides*-induced apoptosis, we analyzed the expression level of this protein. The HepG2 cells treated with *E. alsinoides* for 24 hr showed significant and concentration-dependent (20 and 40 µg (p<0.05); 80 µg (p<0.01)) increases in the expression level of CTNNB1 protein when compared to untreated cells. β-actin was used for normalization of proteins expression levels (Figures 5A and B). 

## Discussion

Natural products are regarded as sources of novel drugs and have served as vital resources of cancer therapeutics (Ezhilarasan, 2018 ▶). Numerous herbal drugs including vinca alkaloids, camptothecin, and paclitaxel have been identified and are still used as cancer chemotherapeutics (Efferth et al., 2017 ▶). Several drugs isolated from herbal plants are currently being tested against various cancers or chemotherapeutics-induced side effects and have shown promising results (Woo et al., 2018 ▶; Tsai et al., 2017 ▶). The efficacy of *E. alsinoides* methanolic extract has not been tested against any cancer cell lines and to the best of our knowledge probably this study is the first one in this regard. 

We demonstrated that *E. alsinoides* methanolic extract can be cytotoxic to HepG2 cells. In a previous study, phytochemicals such as piperine, octadeconoic acids, hexadecanoic acid and squalene have been found in *E. alsinoides* (Gomathi et al., 2015 ▶). Interestingly, piperine from* E. alsinoides*, has been shown to induce growth inhibition and apoptosis in various cancer cell lines (Lin et al., 2014 ▶; Yaffe et al., 2013 ▶; Do et al., 2013 ▶). Taken together, the presence of active phytoconstituents like piperine in *E. alsinoides* extract may contribute to its cytotoxicity observed in HepG2 cells.

Further, to find out the exact reason behind the cytotoxicity, we investigated the apoptosis-related morphological changes upon treatment with *E. alsinoides* in HepG2 cells. Annexin V/PI staining is one of the gold standard methods to morphologically distinguish early and late apoptotic cells (Feng et al., 2018 ▶; Rieger et al., 2011 ▶). Viable cells with intact membranes exclude PI, whereas the membranes of dead and damaged cells are permeable to PI. Therefore, cells that are considered viable are both annexin V and PI negative, while cells that are in early apoptosis are annexin V positive and PI negative, and cells that are in late apoptosis or already dead are both annexin V and PI positive (Rieger et al., 2011 ▶; Baskić et al., 2006 ▶). In view of the above reports, the presence of green colored annexin V stained cells and red colored PI stained cells upon treatment with *E. alsinoides* indicate early and late apoptotic cells, respectively. 

Typical morphological features of apoptotic nuclei such as chromatin condensation and nuclear fragmentation can be better observed through Hoechst staining (Feng et al., 2018 ▶). Apoptotic cells with condensed chromatin can therefore be distinguished from healthy cells or necrotic cells. Hoechst stain has high affinity towards the condensed chromatin (Zhang et al., 2018 ▶). During apoptosis, the DNA becomes condensed, but this process does not occur during necrosis (Zhang et al., 2018 ▶; Feng et al., 2018 ▶). Hoechst staining performed in the present study, demonstrated that HepG2 cells nuclei were altered when compared with controls and the presence of bright fluorescence positive cells upon treatment with *E. alsinoides* indicated nuclear fragmentation and chromatin condensation.

Mitochondrial membrane depolarization is an early event of apoptosis (Sakamuru et al., 2016 ▶) and decreases in the MMP may also be linked to apoptosis. In this study, loss of mitochondrial membrane integrity was observed in the *E. alsinoides*-treated HepG2 cells. Many plant extracts have also been shown to induce apoptosis by reducing MMP (Rajan et al., 2014 ▶; Singh et al., 2011 ▶; Wang et al., 2013 ▶) and our current results are in agreement with the above reports. 

β-catenin is an important intermediate in several signal transduction pathways including the Wnt pathway. It interacts with E-cadherin, a critical regulator of cell-cell adhesion, in plasma membrane (Thakur and Mishra, 2013 ▶). In this study, *E. alsinoides* treatment significantly up regulated the β-catenin protein expression. Studies have shown that deregulation of β-catenin signaling is an important event in the genesis of a number of malignancies, such as colon cancer, melanoma, hepatocellular carcinoma, ovarian cancer, endometrial cancer, medulloblastoma pilomatricomas, and prostate cancer. β-catenin plays an important role in the control of cells proliferation and/or death (Morin, 1999 ▶). However, in recent studies, β-catenin activation is also implicated in cancer progression (Thakur and Mishra, 2013 ▶; Wang et al., 2015 ▶). β-catenin functions as both pro- and anti-apoptotic factor besides inhibiting the recruitment of inflammatory anti-tumor T-cells (Kumar and Bashyam, 2017 ▶). Therefore, β-catenin role in apoptosis is still debated and further studies are warranted.

In conclusion, methanol extract of *E. alsinoides* inhibits HepG2 cell proliferation and triggers apoptotic cell death probably through up-regulation of β-catenin and down-regulation of mitochondrial membrane potential. 
